# Potential of Exosomes for Diagnosis and Treatment of Joint Disease: Towards a Point-of-Care Therapy for Osteoarthritis of the Knee

**DOI:** 10.3390/ijms22052666

**Published:** 2021-03-06

**Authors:** Miki Maehara, Eriko Toyoda, Takumi Takahashi, Masahiko Watanabe, Masato Sato

**Affiliations:** 1 Department of Orthopaedic Surgery, Surgical Science, Tokai University School of Medicine, 143 Shimokasuya, Isehara 259-1193, Kanagawa, Japan; m-maehara@tsc.u-tokai.ac.jp (M.M.); etoyoda@tokai-u.jp (E.T.); ttakahashi@tokai-u.jp (T.T.); masahiko@is.icc.u-tokai.ac.jp (M.W.); 2 Center for Musculoskeletal innovative Research and Advancement (C-MiRA), Tokai University Graduate School of Medicine, 143 Shimokasuya, Isehara 259-1193, Kanagawa, Japan

**Keywords:** exosome, point-of-care therapy, osteoarthritis of the knee, cartilage regeneration

## Abstract

In the knee joint, articular cartilage injury can often lead to osteoarthritis of the knee (OAK). Currently, no point-of-care treatment can completely address OAK symptoms and regenerate articular cartilage to restore original functions. While various cell-based therapies are being developed to address OAK, exosomes containing various components derived from their cells of origin have attracted attention as a cell-free alternative. The potential for exosomes as a novel point-of-care treatment for OAK has been studied extensively, especially in the context of intra-articular treatments. Specific exosomal microRNAs have been identified as possibly effective in treating cartilage defects. Additionally, exosomes have been studied as biomarkers through their differences in body fluid composition between joint disease patients and healthy subjects. Exosomes themselves can be utilized as a drug delivery system through their manipulation and encapsulation of specific contents to be delivered to specific cells. Through the combination of exosomes with tissue engineering, novel sustained release drug delivery systems are being developed. On the other hand, many of the functions and activities of exosomes are unknown and challenges remain for clinical applications. In this review, the possibilities of intra-articular treatments utilizing exosomes and the challenges in using exosomes in therapy are discussed.

## 1. Introduction

Cartilage has poor self-repair and regeneration ability once injured, as first reported in 1743 by William Hunter [1]. Thus, the regeneration of articular cartilage has been a long-standing challenge for physicians and researchers in the field of orthopedics. Articular cartilage consists of highly elastic hyaline cartilage, which is mainly composed of water and extracellular matrix (ECM), such as proteoglycans and collagen fibers. The cellular component of articular cartilage makes up only about 5% of its wet weight and <10% of its tissue volume. Articular cartilage has poor self-renewal ability due to the extremely low infiltration of oxygen, nutrients, and progenitor cells, as it is avascular [2,3].

Osteoarthritis of the knee (OAK) is one of the most common joint diseases. It is a chronic, progressive disease in which the articular cartilage gradually degenerates due to complex interactions between various factors including trauma due to accidents and sports, obesity, skeletal structure, heredity, and age-related overload on the joints. The global prevalence of OAK is estimated to be approximately 3.8%, and it is observed more in women (mean, 4.8%) than in men (mean, 2.8%) [4]. Initially, partial-thickness articular cartilage defects with wear and tear on the cartilage surface are observed. As the pathological condition progresses, the subchondral bone becomes exposed, leading to full-thickness articular cartilage defects. One of the most serious symptoms is pain during daily activities such as walking and climbing the stairs, leading to decreased physical activity and quality of life.

Non-surgical treatments for articular cartilage injury include non-pharmacological treatments such as exercise therapy, weight loss therapy, and orthotic therapy. Pharmacological treatments include utilization of analgesics or non-steroidal anti-inflammatory drugs (NSAID) and intra-articular injection of corticosteroids or hyaluronic acid. Currently, these are the limited options that are widely available for point-of-care treatment for OAK, and they fail to reverse the progress of cartilage degeneration. For patients who show no improvements with non-pharmacological and pharmacological treatments, surgical treatment options are considered.

Surgical options for injuries with small defects include subchondral drilling [5], microfracture [6,7], and mosaicplasty [8,9]. However, the cartilage regenerated through these methods is often fibrocartilage, which is mechanically inferior to the hyaline cartilage that constitutes the original articular cartilage. For larger cartilage defects, autologous chondrocyte implantation (ACI) [10] has been utilized. Currently, products such as JACC^®^ [11,12], CaRes^®^ [13,14], Hyalograft^®^C [15], and BioSeed^®^C [16] are on the market and used worldwide. However, the regenerated cartilage is reported to be either fibrocartilage or a composite of fibrocartilage and hyaline cartilage, and these methods are not yet applicable to OAK [17,18]. In Japan, cell sheet transplantation with high tibial osteotomy [19,20,21] is a cell therapy that has been approved for the treatment of OAK as an advanced medical care. However, the treatment is costly and time consuming. After other options are exhausted, total knee arthroplasty (TKA) is applied. Although the treatment outcomes of TKA are remarkable [22], artificial joints may need revision after 15 to 20 years. Thus TKA is often applied to people over the age of 65.

To bridge the gap between traditional pharmacological and surgical treatments, cell therapies such as mesenchymal stem cells (MSCs) [23,24] and platelet rich plasmas (PRPs) [25,26,27] have been in use as a point-of-care treatment for OAK. Patients can receive these treatments through intra-articular injections at relatively low costs in select places. However, therapeutic effects attributed to various modes of action including cytokines and growth factors can be variable [28,29,30,31]. In addition, risks of inappropriate transformation of the transplanted cells and inflammatory reactions exist [32,33,34,35,36]. Thus, a cell-free therapy that maximizes the therapeutic effect through the selected administration of identified factors is ideal.

To address such issues, extracellular vesicles (EVs), especially exosomes, are attracting attention as a novel tool that can be applied to treat cartilage and bone disease [37,38]. The therapeutic effects of MSCs have also been partially attributed to exosomes carrying specific cargos [39,40,41,42,43]. With recent advancements, exosomes are used as efficient drug delivery systems that can be manipulated to carry specific cargo such as micro ribonucleic acids (miRNAs). As such, exosomes may be used as a point-of-care treatment for joint disease, especially through intra-articular injection. In addition, exosomes may be useful as biomarkers to detect joint disease such as OAK at early onset. More recently, a combination of exosomes with tissue engineering methods has been investigated to prolong and improve the efficacy of exosomes. In this review, we present the recent concepts of exosomes that can be applied to the development of point-of-care treatment for joint disease such as OAK.

## 2. Extracellular Vesicles

Historically, membrane vesicles released by cells have been thought of as non-functional, inactive “debris” resulting from cell damage or turnover of the cellular membrane. However, in 1983, the existence of a 100-nm functional endoplasmic reticulum comprised of lipid bilayer membranes secreted from cells was confirmed [44]. Subsequent research identified membrane vesicles secreted by various cells, which were named according to their features, such as ectosomes, microvesicles, oncosomes, exosomes, and apoptotic bodies [45,46,47,48,49,50,51,52]. To resolve confusion, the International Society for Extracellular Vesicles (ISEV) suggested using the term “extracellular vesicles” (EVs) as “the generic term for particles naturally released from the cell that are delimited by a lipid bilayer membrane and cannot replicate, i.e., do not contain a functional nucleus” [53]. Various subtypes of EVs share similar characteristics, and a method to accurately classify EVs and their fundamental biological roles is unclear [54]. Therefore, the ISEV has suggested a classification of the subtypes of EVs according to the following characteristics: (a) physical characteristics, such as size (“small EVs” (sEVs, <100–200 nm) and “medium/large EVs” (m/lEVs, >200 nm)) or density (low, middle, high); (b) biochemical composition (cluster of differentiation (CD)63+/CD81+ EVs, Annexin A5-stained EVs, etc.); or (c) descriptions of conditions or cell of origin (podocyte EVs, hypoxic EVs, large oncosomes, apoptotic bodies) [53].

## 3. Biogenesis, Composition, and Isolation of Exosomes

The nomenclature of exosomes was suggested in 1987 [46]. Exosomes are a subtype of EVs that have a small diameter of 50–150 nm. They are secreted from most of the cells in the body and are observed in body fluids and cell culture media [47,52].

**Figure 1:**
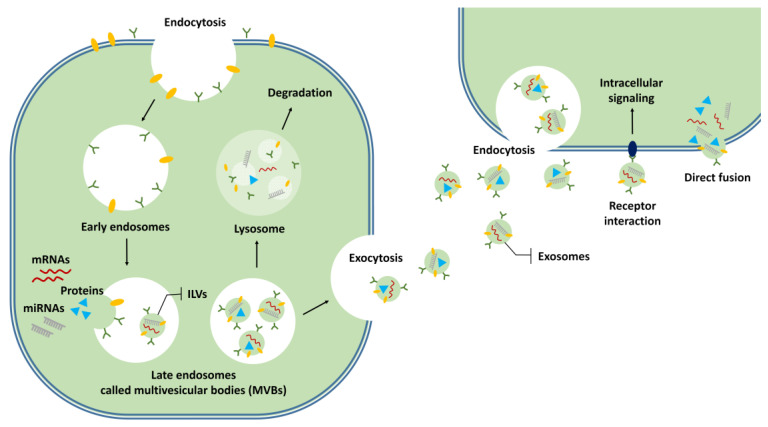
Formation of exosomes and uptake into target cells. Early endosomes are formed by endocytosis and they transition to late endosomes. These are called multivesicular bodies (MVBs), with intraluminal membrane vesicles (ILVs). ILVs that are released from MVBs into the extracellular space are called exosomes. Released exosomes are taken up by the target cells through endocytosis, interactions with receptors on the cell membrane, or direct fusion with the cell membrane.

Exosomes are known to be formed by endocytosis (Figure 1). Early endosomes transition to late endosomes. Then a large number of intraluminal membrane vesicles (ILVs) are formed by internal budding of the endosome membrane. Late endosomes are called multivesicular bodies (MVBs). When the MVBs fuse with the cell membrane, the internal ILVs are released into the extracellular space as exosomes [55] (Figure 1). Endosomal sorting complexes required for transport (ESCRT) and tetraspanins are thought to be involved in the formation of endosomes [47,56,57]. The mechanisms for uptake of the exosomes into target cells and the delivery of the exosome contents are unclear. Many studies have suggested that endosomes are the putative location of EV-content delivery, and contents are released into the cytoplasm of the target cells by fusing with endosome membranes [47,55]. Other mechanisms of content delivery to target cells, such as interaction with the receptors present on the cell membrane and direct fusion with the cell membrane, have also been proposed [47,55].

**Figure 2:**
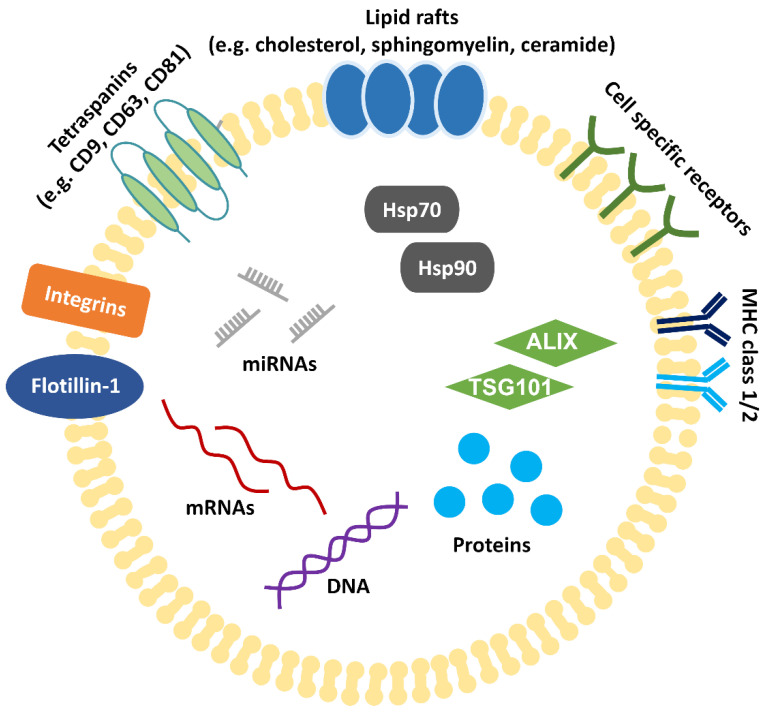
Structure of exosomes. Exosomes have a lipid bilayer membrane composed of lipid raft constituents, tetraspanins (such as CD9, 63 and 81), and proteins (such as integrin, cell specific receptors, MHC class I and class II, and flottilin-1). Exosomes contain cellular components such as proteins, DNA, mRNAs, miRNAs, proteins associated with MVB formation (ALIX, TSG101), and heat shock proteins (Hsp70, Hsp90).

Exosomes are comprised of a lipid bilayer membrane containing lipid raft constituents, such as cholesterol, sphingomyelin, and ceramide, and contain messenger RNAs (mRNAs), miRNAs, deoxyribonucleic acid (DNA), and various proteins (Figure 2) [58,59,60,61]. As of January 2021, 9769 proteins, 1116 lipids, 3408 mRNAs, and 2838 miRNAs have been registered in the ExoCarta database (http://www.exocarta.org/, accessed on 2 January 2021), which catalogues exosome contents. Although various proteins are associated with the lipid membrane of exosomes, no clear biomarker has been identified. Proteins, such as major histocompatibility complex (MHC) class I and class II, heat shock proteins (e.g., heat shock protein (Hsp) 70, Hsp90), flottilin-1, and actin, have been used as “exosome markers” in the past and have been shown to be present in all types of EVs [62]. In addition, the co-expression of tetraspanins (e.g., CD9, CD63, CD81) and proteins associated with MVB formation (e.g., ALG-2-interacting protein X (ALIX), tumor susceptibility gene 101 (TSG101)) have been suggested (Figure 2) [62]. It is not yet clear whether exosomes have specific functions not found in other EVs or whether they can be reliably distinguished and separated from other EVs.

EVs are a heterogeneous population with a mixture of cell vesicles of various fractions. In order to use exosomes for research and treatment, a technique for isolating only “exosome fractions” in large quantities and with high purity is required. Various exosome isolation methods have been developed to this day [63,64,65]. The most classic method is ultracentrifugation. This method is still the most widely used [66] and has become the gold standard for exosome isolation. In addition, there are various methods such as ultrafiltration, polymer precipitation, size exclusion chromatography, immunoaffinity purification, and microfluidics-based techniques. Furthermore, various exosome isolation kits such as exoEasy Maxi kit (QIAGEN, Venlo, Nederland), ExoQuick (System Biosciences, Palo Alto, CA, USA), qEV size exclusion columns (Izon Science, Oxford, United Kingdom), and MagCapture™ Exosome Isolation Kit PS (Wako Pure Chemical corporation, Osaka, Japan) are on the market. Each isolation method has advantages and disadvantages, and the quality and amount of exosomes obtained vary greatly depending on the isolation method selected [63,64,65]. In order to isolate the exosome fraction with the desired activity, it is important to select the optimal exosome isolation method for each objective.

## 4. Potential of Exosomes for Diagnosis and Treatment of Joint Diseases

The potential for exosomes and their derivatives to be delivered through intra-articular injection opens up new possibilities for the treatment of joint diseases such as OAK. Exosomes contain specific information about the cells from which they are released, and may have the ability to deliver molecules to specific organs or tissues at a distance [61,67,68]. Studies have shown that exosomal miRNAs play an important role in joint homeostasis. Furthermore, the imbalances in exosomes created during joint disease can be utilized as diagnostic biomarkers. Studies have also shown the potential for exosomes to be utilized as a drug delivery system (DDS) to deliver specific cargo to localized areas. In combination with tissue engineering, studies have utilized exosomes in a sustained release drug delivery system (SRDDS) to further improve the efficacy. Here, we discuss such topics in regards to their applications to the diagnosis and treatment of joint diseases.

### 4.1. Exosomal miRNAs

MiRNAs are small non-coding RNAs that have approximately 22 nucleotides. These induce gene silencing by sequence-complementary binding to target sites located within the three prime untranslated regions (3’UTR) of the target mRNA and regulate multiple biochemical pathways [69]. Many miRNAs associated with chondrocyte function and homeostasis have been reported to contribute to cartilage repair [70,71]. For example, miR-140, a promising miRNA for OAK treatment, is expressed specifically in articular cartilage and plays an important role in cartilage development and metabolic balance of the cartilage matrix by inhibiting a disintegrin and metalloproteinase with thrombospondin motifs 5 (ADAMTS5) and matrix metalloproteinase 13 (MMP13) [72,73]. Ukai et al. examined cartilage tissues from pediatric patients with polydactylism, young patients with anterior cruciate ligament injury, and elderly patients with OAK and identified miRNA-199a-3p, 193b, and 320c as markers that suppress age-related cartilage metabolism [74]. The discovery that mRNAs and miRNAs are packaged in exosomes that are transported between cells and function in target cells was a breakthrough [59,75,76,77]. Exosomal miRNAs are protected from endogenous RNase activity by the exosome membrane and can exist stably in body fluids and cell culture media. Therefore, exosomal miRNAs can be collected and administered through intra-articular injections. This section introduces the current research on intra-articular treatments with miRNAs (i.e., exosomal miRNAs) secreted extracellularly via exosomes (Table 1).

Studies have shown that exosomal miRNAs may influence cartilage differentiation and homeostasis. Sun et al. performed a microarray analysis of exosomes secreted from both human bone marrow-derived MSCs (hBMSCs) undifferentiated and hBMSCs differentiated into cartilage [78]. Through the comparative analysis, they identified 35 miRNAs, including miR-1246, miR-1290, miR-193a-5p, miR-320c, and miR-92a, that were upregulated in exosomes derived from hBMSCs differentiated into cartilage. In addition, they demonstrated that exosomes derived from miR-320c-overexpressing hBMSCs upregulate SRY-box transcription factor 9 (SOX9) and downregulate MMP13 in OA chondrocytes, and enhance chondrogenesis of hBMSCs [78]. Mao et al. demonstrated that exosomes secreted by OA cartilage express significantly less miR-95-5p compared to normal cartilage [79]. In addition, they showed that exosomes derived from miR-95-5p-overexpressing primary chondrocytes promote cartilage development and cartilage matrix expression by hBMSCs and chondrocytes, and that miR-95-5p may be effective in the treatment of OA by directly targeting histone deacetylase (HDAC) 2/8. Li et al. showed that chondrocyte-derived exosomal miR-8485 promotes the chondrocyte differentiation of BMSCs [80]. The study suggested that miR-8485 may suppress the production of glycogen synthase kinase 3 beta (GSK-3β) by regulating the expression and activity of GSK-3β, activate the Wnt/β-catenin pathway through phosphorylation of GSK-3β by targeting disheveled binding antagonist of beta catenin 1 (DACT1), and promote chondrocyte differentiation.

Other studies have shown the potential of exosomal miRNAs in various animal models. Mao et al. showed that miR-92a-3p directly targets Wnt family member 5A (WNT5A) and promotes proliferation and migration of chondrocytes and the differentiation of MSCs into chondrocytes [81]. In addition, they showed that exosomes derived from miR-92a-3p-overexpressing hBMSCs inhibit the progression of early osteoarthritis (OA) and prevent the severe damage of cartilage in an OA model mouse induced with collagenase VII. Jin et al. found that the expression of miR-26a-5p decreases and the expression of prostaglandin-endoperoxide synthase 2 (PTGS2) increases in OA patients and in synovial fibroblasts treated with interleukin-1 beta (IL-1β) [82]. In addition, they showed that hBMSC-derived exosomes provide miR-26a-5p to synovial fibroblasts and alleviate the damage of OA synovial fibroblasts by controlling PTGS2 expression. Moreover, articular injection of exosomes derived from miR-26a-5p-overexpressing hBMSC into an OA rat model prevents OA damage by alleviating the inflammation of synovial tissues and decreasing apoptosis. Wu et al. demonstrated that infrapatellar fat pad (IPFP) MSC-derived exosomes (MSC^IPFP^-Exos) can deliver miR-100-5p to chondrocytes [83]. The miR-100-5p specifically targets the 3′UTR region of mammalian target of rapamycin (mTOR) and significantly enhances autophagy levels in chondrocytes, inhibits cell apoptosis, enhances anabolism, and represses catabolism in IL-1β-treated OA chondrocytes. In addition, MSC^IPFP^-Exos improved the pathological degree of severity and foot gait patterns in a destabilization of the medial meniscus (DMM)-induced OA mouse model. Tao et al. showed that exosomes derived from synovium-derived MSCs (SMSCs) activate Yes-associated protein (YAP) and promote the proliferation and migration of chondrocytes, but decrease ECM secretion [84]. Therefore, exosomes derived from miR-140-5p-overexpressing SMSCs (SMSC-140-Exos) affected proliferation and migration of chondrocytes without ECM secretion being influenced. Moreover, the administration of SMSC-140-Exos to an anterior cruciate ligament transection (ACLT) OA rat model demonstrated the inhibition of severe damage of the articular cartilage and the progression of OA.

In addition, exosomes are thought to be deeply involved in the development of various physiological phenomena and pathological conditions, and further research on exosomal miRNAs may lead to the elucidation of the mechanism of onset and progression of joint diseases such as OAK. Ni et al. showed that exosome-like vesicles from OA chondrocytes inhibit the expression of autophagy related 4B cysteine peptidase (ATG4B) via miR-449a-5p [85]. This result suggests that inflammation of the synovial membrane may be promoted by the inhibition of autophagy of macrophages and the increase in production of mature IL-1β, leading to progression of OA diseases.

### 4.2. Exosomes as Biomarkers

As OAK patients experience few symptoms during early onset and because of the difficulty in providing treatment as the disease progresses, early diagnostic methods using tools such as biomarkers are desired. Exosomes are anticipated to be useful as novel biomarkers as they contain specific information from the released cells and can stably carry encapsulated substances. In the diagnosis of lung cancer, Exo Dx™ Lung (ALK) (Exosome Diagnostics, Inc., Waltham, MA, USA) is the first Clinical Laboratory Improvement Amendments-validated exosome-based clinical liquid biopsy test that isolates exosomal mRNAs in blood to diagnose cancer [86,87]. For the diagnosis of OAK, exosomes isolated from blood or joint fluid have been identified as useful to date.

For instance, several studies have shown that the expression of specific exosomal miRNAs is altered in patients with joint disease. Kolhe et al. showed that synovial fluid exosomal miRNAs may be altered in OAK patients [88]. In particular, exosomal miRNAs expressed specifically in female OAK patients are responsive to estrogen and target the Toll-like receptor (TLR) signal pathway. Meng et al. confirmed that the expression of exosomal miRNA-193b-3p was decreased in the plasma of OAK patients compared to that of healthy subjects [89].

In addition, Zhao et al. mentioned the possibility that long noncoding RNAs (lncRNAs) can be used as biomarkers for OAK. They conducted an analysis of plasma-derived and synovial fluid-derived exosomal lncRNAs to assess the progression of OAK [90]. As a result, the expression of plasma-derived exosomal lncRNAs showed no significant difference; however, the expression of synovial fluid-derived exosomal lncRNAs was higher in early OA and late-stage OA than in healthy subjects. Specifically, exosomal lncRNA prostate-specific transcript 1 (PCGEM1) was much higher in late OA than in early OA and much higher in early OA than in healthy subjects.

Other studies have examined the use of exosomes as biomarkers to differentiate joint diseases. Chen et al. reported that specific plasma-derived exosomal miRNAs were connected to the common pathogenesis of psoriatic arthritis, psoriasis vulgaris, rheumatoid arthritis (RA), and gouty arthritis. An analysis of the potential target genes revealed that these miRNAs are related to immune disorders and bone injury [91]. In addition, Tsuno et al. demonstrated that serum exosomes of patients with active RA possess different protein profiles compared to patients with inactive RA, patients with OA, and healthy donors [92].

Together, these results indicate that differences in exosome profiles in patients with joint diseases and healthy subjects can be investigated using body fluids such as synovial fluid and plasma, which may lead to the development of various tests and diagnostic methods for OAK and other joint diseases in the future.

### 4.3. Exosomes as a Drug Delivery System (DDS)

**Figure 3:**
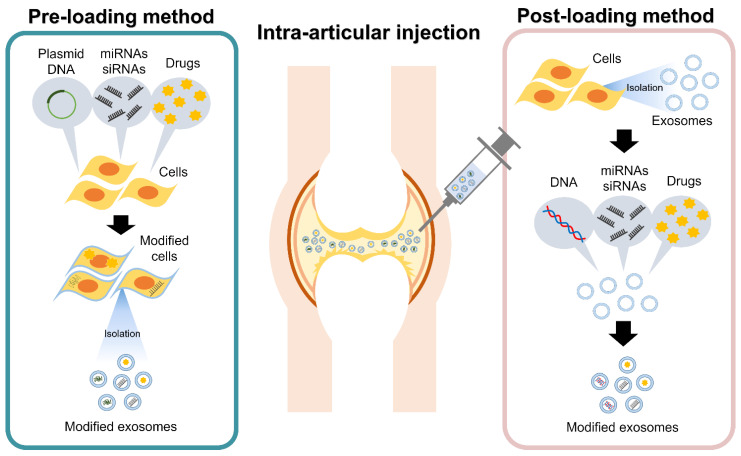
Intra-articular treatments utilizing modified exosomes as a drug delivery system. The loading of specific components into exosomes can be conducted either before exosome isolation (pre-loading method) or after exosome isolation (post-loading method).

If specific miRNAs and other molecules are identified as having a therapeutic effect for the treatment of OAK, they can be packaged in exosomes that can act as a DDS to transport specific contents to target tissues or organs [68,93]. There are two types of methods to enclose the target substances in exosomes: pre-loading and post-loading methods [94] (Figure 3).

Pre-loading methods collect exosomes released by the modified cells after cell modification is performed by miRNA or small interfering RNA (siRNA) transfection or by the uptake of target substances from the culture media during cell culture (Figure 3). Many studies have confirmed the ability of cartilage repair by exosomes collected from cells overexpressing specific miRNAs [71,74,76]. In addition, Wang et al. showed that the expression of miR-135b within the released exosomes can be upregulated by stimulating MSCs using transforming growth factor beta 1 (TGF-β1) [95]. The pre-loading method is a relatively simple procedure that relies on cells to package exosomes. However, it can be difficult to control the loading of specific target molecules in exosomes. A further understanding of the mechanisms by which intracellular miRNAs, mRNAs, and proteins load exosomes efficiently with specific target molecules is necessary.

In contrast, post-loading methods extract exosomes from cells and body fluids, and the target molecules are encapsulated in the exosomes through disruption of the exosome membrane (Figure 3). Post-loading methods include physical induction by electroporation, freeze/thaw method, sonication, etc. and chemical induction by saponin treatment, transfection reagents, etc. In the post-loading method, the target molecules can be directly loaded into the exosomes, so the loading efficiency is expected to be higher than that of pre-loading methods. However, physical disruption may affect the size and shape of exosomes. Chemical induction has been shown to be more efficient in loading target molecules compared to physical induction methods [96]. However, chemicals used for the loading of molecules may be toxic, so removing them in the process is essential.

In terms of applications to joint therapy, post-loading methods may allow the targeting of specific tissues, such as cartilage, subchondral bone, or synovium, to treat the various symptoms. Liang et al. reported on the loading of specific miRNAs through electroporation into engineered exosomes [97]. They engineered chondrocyte-affinity peptide (CAP) exosomes by fusing CAP with lysosome-associated membrane glycoprotein 2b (Lamp2b) on the surface of exosomes. Furthermore, they loaded miR-140 into the CAP exosomes using electroporation (CAP exosome/miR-140). The CAP exosomes/miR-140 allowed for the specific delivery of chondrocytes. Moreover, in a DMM-induced OA rat model, they demonstrated that the progression of OA can be reduced by CAP-exosome/miR-140. Through modification of exosomes and post-loading methods, they were able to simultaneously achieve tissue-specific delivery and inclusion of target molecules into exosomes. These improvements in exosome modification technology could enhance the effectiveness of exosome-based intra-articular treatments.

### 4.4. Potential of Exosomes in a Sustained Release Drug Delivery System (SRDDS)

Exosomes are relatively stable in vivo, but multiple administrations may be required to overcome the natural elimination of exosomes in the joint. As such, to prolong and enhance the effect of joint treatment methods, studies have combined exosomes with various biomaterials to be used in a SRDDS [98,99,100,101]. Chen et al. produced a 3D-printed cartilage ECM/gelatin methacrylate (GelMA)/exosome scaffold with radially oriented channels through a desktop-stereolithography technology [102]. This 3D printed scaffold was shown to continuously release exosomes for 14 days in vitro. When the 3D printed scaffold was transplanted through an invasive procedure in a rabbit osteochondral model, the scaffold retained exosomes for at least 7 days in vivo and significantly promoted cartilage repair.

Other studies have combined exosomes with injectable materials that can be administered through intra-articular injection. Liu et al. utilized stem cell-derived exosomes in combination with photoinduced imine crosslinking hydrogel glue to produce a cell-free exosome tissue patch [103]. The tissue patch continuously released exosomes for 14 days. In vivo studies showed that the new cartilage tissue regenerated by the tissue patch was hyaline cartilage-like in a rabbit articular cartilage defect model. Alternatively, Hu et al. produced a Gelma/nanoclay hydrogel (Gel-nano-sEVs) with embedded human umbilical cord MSC-derived small extracellular vesicles (hUC-MSCs-sEVs) [104]. In this study, they showed that miR-23a-3p, which is highly expressed in hUC-MSCs-sEVs, activates the phosphatase and tensin homolog deleted from chromosome 10 (PTEN)/AKT serine/threonine kinase (AKT) signaling pathway and promotes cartilage regeneration. The Gel-nano-sEVs were demonstrated to release exosomes continuously for approximately 30 days and contribute to cartilage regeneration. Further studies are expected in this field.

## 5. Advantages and Challenges of Developing Treatments Utilizing Exosomes

Treatments utilizing exosomes have remarkable potential to target and affect specific tissues. With proper control, specific cargos such as miRNAs can be packaged to be delivered to various tissues affected by OAK, such as cartilage, synovium, and subchondral bone. Unlike cell-based therapies, exosomes have a lower immunogenicity and an inability to directly form tumors [105,106]. Cell-based therapies rely on a more complicated mode of action such as the production of humoral factors that may be influenced by local conditions [34,107]. While exosomes may also be influenced by local conditions as well, with proper manufacturing they can carry and deliver a consistent set of cargos at desired concentrations.

On the other hand, many challenges remain, such as the large number of cells required for the manufacturing of exosomes, the multiple steps and time required for collection, and the unclear risks in ensuring safety and efficacy [39,41,43,55]. In addition, when exosomes are used clinically, the possible transmission of infectious diseases, due to contamination with viruses, bacteria, and fungi, harmful effects of components other than exosomes that are simultaneously administered, and potential risks of inconsistent efficacy and quality must be considered.

Exosomes possess complicated characteristics such as their size, the cargos they hold, and their destinations, which must be properly characterized in addition to the origins of the exosomes. As such, setting quality standards in the manufacturing process will be a challenge. When manufacturing exosomes for treatments, the quality of cells used as raw materials and their constituents and the management of the manufacturing process will be important additions to the quality control of the exosomes themselves. The ISEV proposed Minimal Information for Studies of Extracellular Vesicles (MISEV) guidelines in 2014, which was updated in 2018, suggesting a comprehensive yet evolving guideline for studies using extracellular vehicles, including exosomes [53,108]. To secure the quality, efficacy, and safety of exosomes, strict quality control and manufacturing control must be implemented.

## 6. Conclusions

EVs, including exosomes, present new possibilities for treatment, diagnosis, and drug development for patients with joint diseases including OAK. Exosomes from body fluids such as joint fluid and plasma can provide important prognoses of disease to administer appropriate treatments and other preventive measures. The use of exosomes for treating OAK through intra-articular injection provides a novel treatment method to bridge the gap between pharmacological and surgical procedures. However, many unclear aspects of the functions and mechanisms of EVs, including exosomes, and their roles in treatments of joint diseases such as OAK, remain to be elucidated. For the realization of exosome therapy, it is essential to clarify the risks of the treatment methods, implement strict manufacturing controls, and prepare appropriate guidelines and laws.

Further research in this field and scientific evidence of safety and efficacy may greatly contribute to the point-of-care treatment and cell-free therapy of joint disease.

## Figures and Tables

**Table 1:** Studies on exosomal miRNAs regulating chondrocyte function and homeostasis for the purpose of intra-articular treatments.

Exosome Origin Cell Source	Study Design	Animal Models	miRNA	Result(s)	Target(s)	Reference
Human BMSCs	in vitro	-	miR-320c	Upregulates SOX9 and downregulates MMP13 expression in OA chondrocyte.	Not mentioned	[78]
Human BMSCs	in vitro	-	miR-95-5p	Enhances histone H3 acetylation and maintains the function of articular chondrocytes. Promotes SOX9, COL2A1 and Aggrecan expression and enhances cartilage development.	HDAC2/8	[79]
Human chondrocytes	in vitro	-	miR-8485	Activates Wnt/β-catenin pathways. Promotes chondrogenic differentiation of hBMSCs.	GSK3B, DACT1	[80]
Human BMSCs	in vitro and in vivo	Mice	miR-92a-3p	Promotes cartilage proliferation. In both MSCs and PHCs, promotes matrix genes expression and inhibits WNT5A expression.	WNT5A	[81]
Human BMSCs	in vitro and in vivo	Rats	miR-26a-5p	It was shown that the damage of synovial fibroblasts is suppressed in vitro, and the OA damage is alleviated in vivo.	PTGS2	[82]
Human IPFP MSCs	in vitro and in vivo	Mice	miR-100-5p	Protects cartilage from damage and ameliorate gait patterns of DMM-induced OA mice.	mTOR	[83]
Human SMSCs	in vitro and in vivo	Rats	miR-140-5p	Enhances the proliferation and migration of ACs, and the progression of early OA and prevents severe damage to knee articular cartilage in the OA rats.	RalA	[84]

hBMSC: human bone marrow-derived MSC, IPFP: infrapatellar fat pad, SMSC: synovium-derived MSC, MSC: mesenchymal stem cell, PHC: primary human chondrocytes, WNT5A: Wnt family member 5A, OA: osteoarthritis, SOX9: SRY-box transcription factor 9, MMP13: matrix metalloproteinase 13, COL2A1: Collagen type II alpha 1, DMM: destabilization of the medial meniscus, AC: articular cartilage, PTGS2: Prostaglandin-endoperoxide synthase 2, HDAC: histone deacetylase, mTOR: mammalian target of rapamycin, RalA: Ras-related protein, GSK-3β: glycogen synthase kinase 3 beta, DACT1: dishevelled binding antagonist of beta catenin 1.

## Abbreviation

3′UTR	three prime untranslated region
ACI	autologous chondrocyte implantation
ACLT	anterior cruciate ligament transection
ADAMTS5	a disintegrin and metalloproteinase with thrombospondin motifs 5
AKT	AKT serine/threonine kinase 1
ALIX	ALG-2-interacting protein X
ATG4B	autophagy related 4B cysteine peptidase
CAP	chondrocyte-affinity peptide
CD	cluster of differentiation
DACT1	dishevelled binding antagonist of beta catenin 1
DDS	drug delivery system
DMM	destabilization of the medial meniscus
DMOAD	disease-modifying OA drugs
DNA	deoxyribonucleic acid
ECM	extracellular matrix
ESCRT	endosomal sorting complexes required for transport
EV	extracellular vesicle
GelMA	gelatin methacrylate
GSK-3β	glycogen synthase kinase 3 beta
hBMSC	human bone marrow-derived MSC
HDAC	histone deacetylase
Hsp	heat shock protein
hUC	human umbilical cord
IL-1β	interleukin-1 beta
ILV	intraluminal membrane vesicle
IPFP	infrapatellar fat pad
ISEV	International Society for Extracellular Vesicles
Lamp2b	lysosome-associated membrane glycoprotein 2b
lncRNA	long noncoding RNA
MCH	major histocompatibility complex
MCS	mesenchymal stem cell
miRNA	microRNA
MISEV	Minimal Information for Studies of Extracellular Vesicles
MMP13	matrix metalloproteinase 13
mTOR	mammalian target of rapamycin
MVB	multivesicular body
NSAID	non-steroidal anti-inflammatory drugs
OAK	osteoarthritis of knee
PCGEM1	prostate-specific transcript 1
PHC	primary human chondrocytes
PTEN	phosphatase and tensin homolog deleted from chromosome 10
PTGS2	prostaglandin-endoperoxide synthase 2
RA	rheumatoid arthritis
RNA	ribonucleic acid
siRNA	small interfering RNA
SMSC	synovium-derived MSC
SOX9	SRY-box transcription factor 9
SRDDS	sustained release drug delivery system
TGF-β1	transforming growth factor beta 1
TKA	total knee arthroplasty
TLR	Toll-like receptor
TSG101	tumor susceptibility gene 101
WNT5A	Wnt family member 5A
YAP	Yes-associated protein
